# Altered sensorimotor processing in irritable bowel syndrome: Evidence for a transdiagnostic pathomechanism in functional somatic disorders

**DOI:** 10.3389/fnins.2022.1029126

**Published:** 2022-11-09

**Authors:** Lena Schröder, Franziska Regnath, Stefan Glasauer, Anna Hackenberg, Juliane Hente, Sonja Weilenmann, Daniel Pohl, Roland von Känel, Nadine Lehnen

**Affiliations:** ^1^Department of Psychosomatic Medicine and Psychotherapy, Klinikum rechts der Isar of the Technical University of Munich, Munich, Germany; ^2^Computational Neuroscience, Institute of Medical Technology, Brandenburg University of Technology Cottbus-Senftenberg, Senftenberg, Germany; ^3^Graduate School of Systemic Neurosciences, Ludwig-Maximilians-Universität München, Planegg, Germany; ^4^Department of Sport and Health Sciences, TUM Graduate School, Technical University of Munich, Munich, Germany; ^5^Faculty of Health Sciences Brandenburg, Brandenburg University of Technology Cottbus-Senftenberg, Senftenberg, Germany; ^6^Department of Gastroenterology and Hepatology, University Hospital Zurich, University of Zurich, Zurich, Switzerland; ^7^Department of Consultation-Liaison Psychiatry and Psychosomatic Medicine, University Hospital Zurich, University of Zurich, Zurich, Switzerland; ^8^Insititute of Medical Technology, Brandenburg University of Technology Cottbus-Senftenberg, Senftenberg, Germany

**Keywords:** irritable bowel syndrome (IBS), functional somatic disorders, somatoform disorders, predictive processing, transdiagnostic mechanism, gaze shift

## Abstract

**Objective:**

A recent hypothesis suggests that functional somatic symptoms are due to altered information processing in the brain, with rigid expectations biasing sensorimotor signal processing. First experimental results confirmed such altered processing within the affected symptom modality, e.g., deficient eye-head coordination in patients with functional dizziness. Studies in patients with functional somatic symptoms looking at general, trans-symptomatic processing deficits are sparse. Here, we investigate sensorimotor processing during eye-head gaze shifts in irritable bowel syndrome (IBS) to test whether processing deficits exist across symptom modalities.

**Methods:**

Study participants were seven patients suffering from IBS and seven age- and gender-matched healthy controls who performed large gaze shifts toward visual targets. Participants performed combined eye-head gaze shifts in the natural condition and with experimentally increased head moment of inertia. Head oscillations as a marker for sensorimotor processing deficits were assessed. Bayes statistics was used to assess evidence for the presence or absence of processing differences between IBS patients and healthy controls.

**Results:**

With the head moment of inertia increased, IBS patients displayed more pronounced head oscillations than healthy controls (Bayes Factor _10_ = 56.4, corresponding to strong evidence).

**Conclusion:**

Patients with IBS show sensorimotor processing deficits, reflected by increased head oscillations during large gaze shifts to visual targets. In particular, patients with IBS have difficulties to adapt to the context of altered head moment of inertia. Our results suggest general transdiagnostic processing deficits in functional somatic disorders.

## Introduction

Recently, it was hypothesized that functional somatic symptoms, i.e., debilitating physical symptoms in the absence of a sufficiently explaining organic deficit, emerge, and manifest as a result of erroneous sensorimotor processing (Edwards et al., 2012; Van den Bergh et al., 2017; Henningsen et al., 2018; Pezzulo et al., 2019). This theory is based on predictive processing, a neurobiological framework describing normal brain function (Srinivasan et al., 1982; Mumford, 1992; Rao and Ballard, 1999; Friston, 2002; Knill and Pouget, 2004; Aitchison and Lengyel, 2017). The brain constantly manages situations in which sensory input is ambiguous or noisy (“perceptual problem,” first described in von Helmholtz, 1867) by integrating prior knowledge that anticipates sensory information into the perceptual process. Expectations derived from central nervous system (CNS)-internal models representing learned causal relationships in the world and within the body are tightly interwoven with information provided by body sensors already at low hierarchical levels in the brain (Shams and Beierholm, 2010; Hohwy, 2013). This leads to perceptions and actions that always include the product of both, prior knowledge and sensory input. Adaptive behavior in a rapidly changing environment is only possible when this interaction is highly flexible: new situations require more focus on sensory input, as prior knowledge about the situation is still lacking, while during well-known situations, it is beneficial to rely on (successfully acquired) knowledge from the past rather than considering each sensory fluctuation (Clark, 2013; Rauss and Pourtois, 2013). In the case of functional somatic symptoms, it is now assumed that this fine-tuned information processing system is out of balance, so that rigid expectations dominate sensory input during sensorimotor processing, leading to symptom perception (Edwards et al., 2012; Van den Bergh et al., 2017; Henningsen et al., 2018; Pezzulo et al., 2019).

There is experimental evidence for such processing deficits in functional somatic symptoms and disorders (Bogaerts et al., 2010; Van Den Houte et al., 2017; Lehnen et al., 2019; Schröder et al., 2021). In an eye-head coordination paradigm (first described in Lehnen, 2006), patients with functional dizziness showed poorer head motor control compared to healthy controls. Patients displayed stronger head oscillations at the end of a gaze shift, reflecting adaptation deficits in sensorimotor processing, possibly due to incorrect internal expectations (Lehnen et al., 2019). Another study found that gaze movements are also unstable during such large gaze shifts in patients with functional dizziness (Schröder et al., 2021). This was only observed in situations where prior knowledge and sensory information interacted with each other, not during purely sensory-driven stabilization. Taken together, these two studies provide evidence for erroneous internal model/expectation use in sensorimotor processing in functional dizziness. Here, erroneous processing is directly linked to the symptom modality, i.e., the vestibular system for gaze motor control. Similarly, Bogaerts et al. (2010) investigated perception of breathlessness in patients with functional dyspnea and healthy controls. After experimental induction of breathlessness by increasing the carbon dioxide (CO_2_) concentration in the inhaled air, patients reported sustained breathlessness even after CO_2_ levels had normalized again. Symptom perception was uncoupled from sensory input and was explained by the influence of prior knowledge altering sensorimotor processing within the perceptual process.

Interestingly, in the described re-breathing paradigm, characteristic alterations in symptom perception were not only found in patients with functional dyspnea, but also for patients with other functional somatic disorders, i.e., fibromyalgia and chronic fatigue syndrome (Van Den Houte et al., 2018). This raises the question whether there are generally transdiagnostic alterations in sensorimotor processing in all functional somatic disorders. To explore this research question in more depth, we applied the gaze shift paradigm, which had previously revealed processing deficits in functional dizziness, to patients with irritable bowel syndrome (IBS). So far, no experimental studies have demonstrated sensorimotor processing deficits in patients with IBS. With its symptoms arising predominantly in the lower gastrointestinal tract, the clinically relevant symptoms of IBS may not directly be linked to the gaze motor control system and therefore suitable to study general sensorimotor processing deficits across organ systems. The gaze shift paradigm investigates head oscillations during gaze shifts under increased head moment of inertia as a marker for sensorimotor processing deficits. This has been demonstrated in patients with functional dizziness (Lehnen et al., 2019), patients with cerebellar ataxia (Sağlam et al., 2014), and patients with bilateral vestibulopathy (Lehnen et al., 2009; Sağlam et al., 2014). Importantly, these studies show that within one single head movement, both, correct vestibular processing as well as intact feedforward prediction are necessary to reduce head oscillations under increased head moment of inertia. This is different from predictability in motor learning. In line with the findings from Van Den Houte et al. (2018), we assumed that general symptom-unspecific processing deficits are present in functional somatic disorders. Specifically, we hypothesized that when experimentally subjected to increased head moment of inertia, patients with IBS will show higher head oscillations than healthy controls.

## Materials and methods

### Participants

For this experimental study, seven patients suffering from IBS [age 33 ± 11, mean and standard deviation (SD), 4 women] and seven age- and gender-matched healthy controls (age 33 ± 13, mean and SD, 4 women) were included. *A priori* sample size estimation in a power analysis (α = 0.05, β = 0.8) based on group differences in our previous studies on functional dizziness (partial η^2^ = 0.62, Lehnen et al., 2019) revealed three participants required for each group. Due to this small number, we increased sample size gradually and used Bayesian statistics that allows for stopping testing when data gives sufficient support for the hypothesis (Wagenmakers et al., 2012; Rouder, 2014).

Patients were recruited from a specialized outpatient clinic for Neurogastroenterology and Motility of the University Hospital Zurich as well as the in- and outpatient clinic of the Department of Psychosomatic Medicine and Psychotherapy of the University Hospital of the Technical University Munich. All patients fulfilled the diagnostic criteria of somatoform autonomic dysfunction of the lower gastrointestinal tract according to ICD-10, which was the inclusion diagnosis (F45.32, World Health Organization, 2004). After Rome IV criteria (Rome Foundation, 2016), six patients fulfilled the diagnosis of an IBS, one patient had functional constipation. After S3-guidelines, all patients had IBS (Layer et al., 2021; for a detailed description of the clinical characteristics of this patient group, see Table 1). Importantly, for all patients, previous gastrointestinal workups including a colonoscopy did not reveal any organ pathology accounting for the patients’ symptoms. Patients did not have any other persisting somatic symptoms corresponding to a somatic symptom disorder, as assessed with the structural clinical interview for DSM-5 on the day of the study (German version of the SCID-5-CV; Beesdo-Baum et al., 2019).

**TABLE 1:** Description of patients’ characteristics.

	Symptom occurrence			Symptom types					
		
Patient	Onset (years)	Frequency (days/week)	Duration (hours)	Abdominal pain	Cramps	Diarrhea	Obstipation	Bloating	Flatulence
1	4	7	12	x		x		x	
2	6	3	0.5–24	x	x	x		x	x
3	1	7	6	x		x		x	x
4	10	7	24		x	x		x	x
5	5	3	5–8	x	x		x		
6	4	3	24	x				x	
7	2	7	3		(x)	(x)	x	x	x

The table provides an overview about symptom criteria of included patients with IBS. Symptom onset describes how many years ago the symptoms first appeared, symptom frequency describes how often symptoms occur on average during the week and duration describes the average time of symptom presence during the day. The type of reported abdominal symptoms by each patient is also shown.

Healthy controls were recruited from the staff of the University Hospital of the Technical University Munich as well as the staff of medical practices and student groups around Munich. On the day of study conduction, they did not fulfill the criteria of a psychiatric disorder according to the German version of the SCID-5-CV (Beesdo-Baum et al., 2019), and, in particular, did not report any current or previous persisting somatic symptoms of functional nature.

All participants had no history of balance disorders. Additionally, to test for an intact vestibular system on the day of study conduction, we performed a video-assisted head impulse test (vHIT) after the vHIT manual of EyeSeeCam (EyeSeeTec GmbH, Munich, Germany), which was normal in patients as well as healthy controls.

The study was designed in line with the Declaration of Helsinki (version from 2008). The Ethics Committee of the Technical University Munich approved the study protocol prior to study conduction. The Ethics Commission of the Kanton Zurich stated that no additional approval was necessary, as study and data responsibility was in Munich alone. All participants provided written informed consent and received a compensation of 10€ per hour.

The current study is part of the innovative training network ETUDE (Encompassing Training in functional Disorders across Europe; https://etude-itn.eu/; see Rosmalen et al., 2021), ultimately aiming to improve the understanding of mechanisms, diagnosis, treatment and stigmatization of Functional Disorders.

### Experimental task

Participants were seated in front of a desk, where five light emitting diodes (LEDs) were placed at eye level in the vertical plane. In the horizontal plane, one LED was placed in front of participant’s head, two LEDs were placed on each side, left, and right, in 70 and 83 cm distance to the central LED. Then, the seating position was adjusted so that the distance from participant’s eyes to the central LED amounted to 1 m. In consequence, from the participant’s perspective, gaze shifts toward flashing LEDs corresponded to 0°, 35°, 70°, 75°, and 80° amplitude. During one experimental round, target light was presented 52 times, requiring 52 gaze shifts in total. It is important to note that the target was off during gaze movement to avoid instantaneous visual feedback. To achieve this, LEDs were flashed in complete darkness for less than 0.1 s. The time interval between the light flashes (1.6–2.4 s) as well as their order were randomized to prevent anticipation.

**FIGURE 1:**
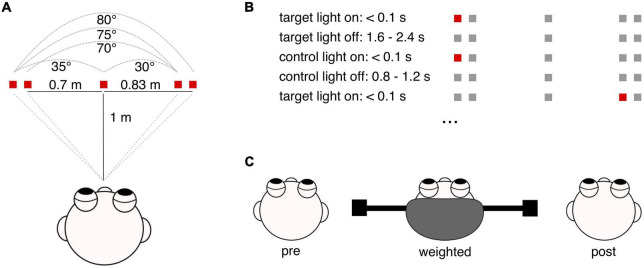
Graphical illustration of the experimental paradigm. **(A)** Shown is the experimental setup in the horizontal plane, with the distances of LEDs to each other and to the participant’s middle head position as well as the resulting gaze shift amplitudes. **(B)** Depiction of the experimental paradigm. For illustration of the experimental timing, an exemplary sequence of target and control lights is presented. Each trial, i.e., one gaze shift towards the target, starts with target flash for less than 0.1 s. This is followed by a sequence of 1.6–2.4 s where the target light is off again. During this period, i.e., without visual input, participants perform a gaze shift towards the target. Then, a control light is flashed at target position, also for less than 0.1 s. In the following period of 0.8–1.2 s, with the control light being off, participants can adjust final gaze position depending on the feedback they received by the control light. The next target flash presents the start of the next trial, requiring another gaze shift (here, the gaze shift amounts to 75°). In total, participants performed 52 gaze shifts. **(C)** Depicted is the order of the three experimental rounds: pre, weighted and post-condition. The drawing illustrates the construction of the experimental helmet, with masses being attached eccentrically at each side.

We instructed participants to direct their gaze to the flashing LEDs in a natural manner, using combined eye-head movements. Once final gaze position was achieved, participants were asked to hold their gaze stable until the next target was flashed. To ensure that participants hold final gaze position, after the actual gaze shift, a control light was flashed at target position, with a 0.8–1.2 s time window before the next target light appeared (Figure 1). Participants were told that they may use the second flash as feedback to adjust gaze position.

Participants performed three rounds of this experimental task. First, 52 gaze shifts were performed in the natural condition (pre). Then, we increased participants’ head moment of inertia to the 3.1-fold by using a specially designed helmet with eccentrically placed masses on the left and right side. After executing all 52 gaze shifts with the helmet (weighted), participants completed a third round of the experiment without the helmet again (post). All participants had no experience in wearing the helmet and were naïve to the experimental hypotheses.

We recorded participants’ eye and head movements with the EyeSeeCam measuring system (EyeSeeTec GmbH, Munich, Germany). The system uses video-oculography to track eye movements and 3D inertial sensors to track head movements with a sampling rate of 220 Hz. The camera was adjusted to record movements of the left eye, the inertial sensors were attached between both eyes in the middle of the forehead.

### Data analysis

Data analysis was conducted offline using MATLAB (MathWorks, Natick, MA, United States). To investigate head movements as part of large horizontal gaze shifts toward visual targets, head velocity in the horizontal plane was obtained from the 3D inertial sensor recordings of the EyeSeeTec measuring system. Head data was then filtered with a 20 Hz Gaussian low pass filter. To estimate the amplitude of the whole eye-head gaze shift, head velocity was further integrated over time to estimate head position. Eye position in the horizontal plane was computed from pupil rotation vectors and was also filtered with a 20 Hz Gaussian low pass filter. Gaze position in space was then computed as the sum of the eye and head position, as eye position was measured in relation to the head and head position was measured in relation to space. Subsequently, the continuous filtered eye, head, and gaze data streams were cut into single trials, so that each movement sequence corresponded to one gaze shift. Trial start was defined as the onset of the target light, trial end was defined as the onset of the control light. Head movements were analyzed during the actual gaze shift period toward the target; possible small corrections of gaze position after presenting the control light were not evaluated in this analysis. Of all 52 gaze shifts, only gaze shifts with a target amplitude of 75° or 80° were considered for the analysis, resulting in 43 valid trials. Furthermore, only gaze shifts with an executed amplitude of at least 40° were included in the analysis.

For each trial, head oscillations were assessed according to Lehnen et al. (2019) and computed as the first undershoot of head velocity at the end of the active head movement toward the target, normalized by peak velocity of the head movement. This was implemented by detecting the maximum of head velocity during the whole trial and the minimum of head velocity between the first zero crossing (head velocity undershoots and becomes negative) and the second zero crossing (head velocity becomes positive again, the first oscillation is terminated). The absolute value of the undershoot was then divided by peak head velocity. Head oscillations were detected automatically and, in case detection errors were identified during visual inspection, were corrected manually. In total, in 5% of healthy controls’ gaze shifts and 10% of the patients’ gaze shifts, detected maxima and minima were corrected. Only trials where the peak head velocity as well as the velocity of the undershoot could be detected were considered for the analysis.

In case of a predictive response, i.e., participants performed the gaze shift before the target light was flashed, the movement window before the actual target light presentation was included into the analysis (on average 3.3% of the gaze shifts for patients and 0.5% for healthy controls). This was done to include as many gaze shifts as possible. Similarly, if a gaze shift was executed delayed, i.e., when head oscillation was not terminated before the control light was presented, the movement window after the target light window was added to the analyzed movement sequence, affecting 6.2% of the trials for patients and 3.3% of the trials for healthy controls.

Head oscillations were computed for each of the three experimental rounds (pre, weighted, and post condition). In a subsequent outlier analysis, head oscillations outside the range of 2 SDs from the mean of the respective subject and condition were removed from the analysis. On average, for every experimental round 36 ± 5, 36 ± 9, and 36 ± 6 of the 43 trials per condition were considered for the IBS patients and 38 ± 4, 40 ± 1, and 40 ± 2 for the healthy control group for the three experimental conditions pre, weighted and post, respectively.

### Statistical analysis

Data were analyzed offline using MATLAB (MathWorks, Natick, MA, United States) and JASP (JASP Team, 2019, Version 0.15).^1^ Mean values of head oscillations were computed for each participant and each condition. Shapiro Wilk test was used to test for normality assumption in all groups and conditions, with a significance level of *p* = 0.05. For hypothesis testing, a Bayes repeated measures ANOVA (rmANOVA) was computed to test for differences in head oscillation between patients with IBS and healthy controls (between-factor *group*) for the three experimental rounds (pre, weighted, post; within-factor *weight*). For *post hoc* comparisons, Bayesian dependent and independent *t*-tests were computed. Bayesian statistics was used because of its possibilities to find evidence for the null hypothesis (Rouder et al., 2007; Wagenmakers, 2007), and to evaluate evidence during accumulation so that testing can be stopped when sufficient support for a hypothesis is given (Wagenmakers et al., 2012; Rouder, 2014).

In a Bayes rmANOVA, all measuring factors as well as their combinations and interactions are considered as models to explain the dataset (see e.g., Wagenmakers et al., 2018; van Doorn et al., 2021). Before testing, the models are assigned with the same prior probability, so that all models are equally likely before seeing the data. In our scenario, with one repeated measures factor and one group factor, there are five possible models to explain the data (*weight*, *group*, *weight* + *group*, *weight* + *group* + weight*group, null model), so each model receives a prior probability of 0.2. Then, using Bayes statistics, the posterior probability is computed, indicating how likely a model is given the data. The model which fits best to the data receives the greatest proportion of posterior probability. Therefore, the posterior probability provides the most relevant output in terms of evaluating evidence of models/effects. To compare models, a Bayes Factor (BF) is computed that shows the ratio between the posterior probabilities of two models. For hypothesis testing, the posterior probability of a model is typically compared to the null model (BF_10_). BF_10_ indicates how many times the model explains the data better than the null model. A BF_10_ of 1 shows that the posterior probability of the null model and the model are the same, so no evidence for the presence or the absence of an effect is given. With increasing BF_10_, it becomes more and more likely that an effect is present. Conventions evaluate a BF_10_ between 1 and 3 as anecdotal evidence, between 3 and 10 as moderate evidence, between 10 and 100 as strong evidence, and above 100 as extreme evidence for the presence of an effect (Wagenmakers et al., 2018). Importantly, as the null model is assigned with a posterior probability, the BF_10_ can also show evidence for the absence of an effect (Rouder et al., 2007; Wagenmakers, 2007). BF_10_ between 1/3 and 1 is evaluated as anecdotal evidence, between 1/3 and 1/10 as moderate evidence, between 1/10 and 1/100 as strong evidence and below 1/100 as extreme evidence in favor of the null hypothesis.

For comparability with our previous studies, we also computed a frequentist rmANOVA to assess differences in head oscillations over the three experimental rounds (pre, weighted, post; within-factor *weight*) and between patients with IBS and healthy controls (between-factor *group*). For *post hoc* comparisons, dependent and independent *t*-tests with Bonferroni corrected α-levels were computed.

## Results

Group analysis with a Bayesian rmANOVA revealed that the model which included the factor *weight*, the factor *group* as well as their interaction was most likely given the data (*weight* + *group* + weight*group: BF_10_ = 3.4*10^10^, corresponding to an extreme effect). To look at the contribution of each factor to explain the data, effect sizes were computed. They estimate the likelihood of models in which the factor was included in comparison to models in which the factor was excluded. The BF for inclusion (BF_*incl*_) of the factor *weight* was 2.9*10^10^, BF_*incl*_ for *group* was 7 and BF_*incl*_ for the weight*group interaction was 5.4, demonstrating extreme evidence for *weight* and moderate evidence for *group* and their interaction.

**FIGURE 2:**
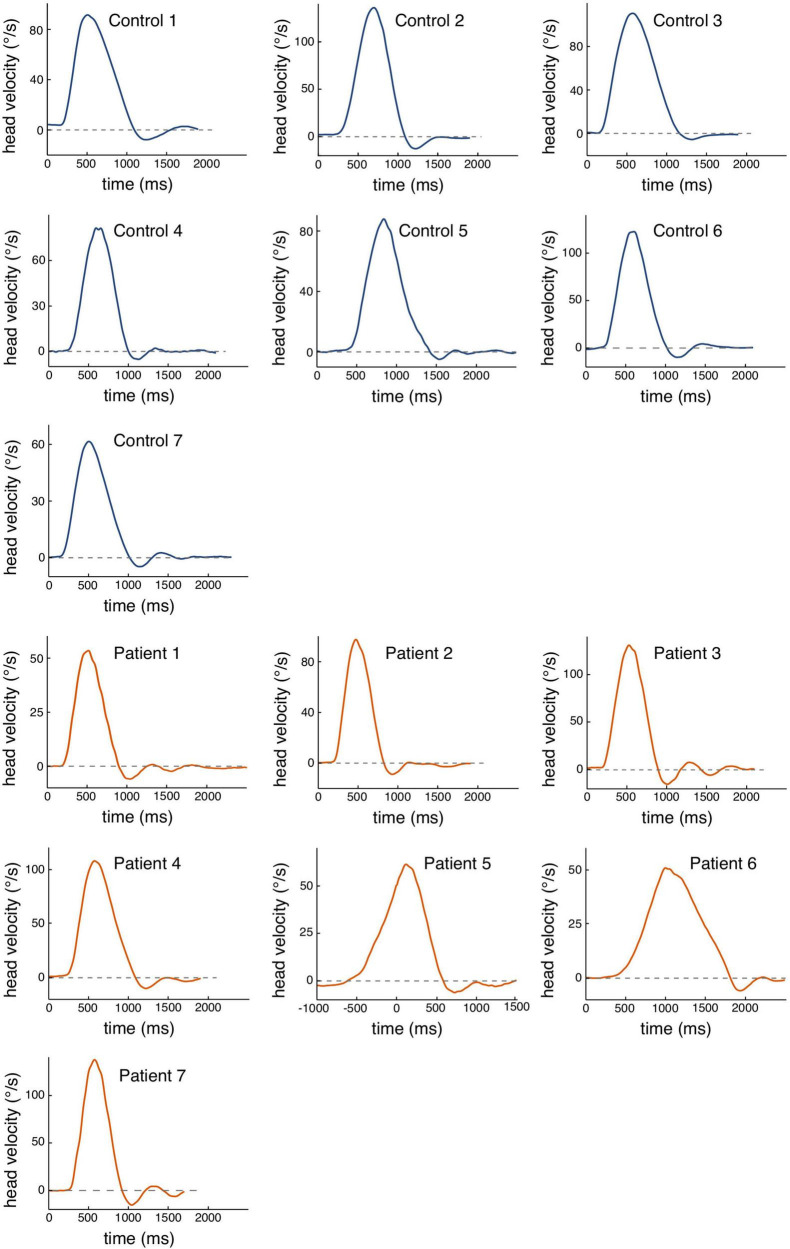
Raw data of head velocity profiles for each participant for the weighted condition. The traces represent one head movement toward the target with an oscillation and peak head velocity value that represents the mean of the respective subject. Blue traces are exemplary movements for healthy controls, orange traces show movements for each IBS patient. The dashed line represents the zero line.

**FIGURE 3:**
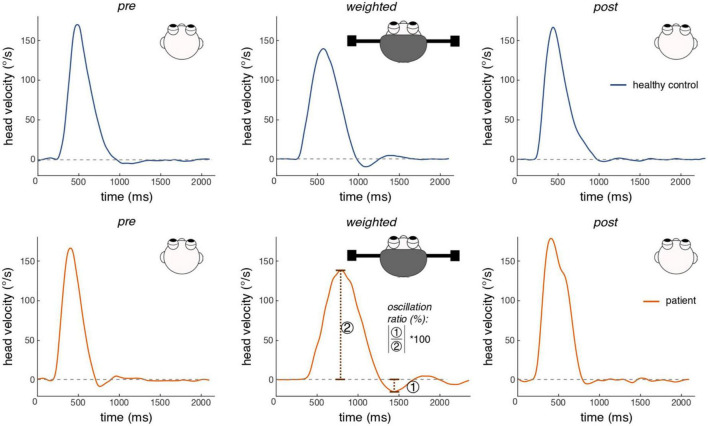
Exemplary head movements of one healthy participant and one patient for each experimental condition. Shown are head velocity profiles of one gaze shift toward a flashing visual target in 75° horizontal distance. Blue traces are exemplary movements of a healthy participant, orange traces are exemplary movements of one patient. Traces were selected to illustrate mean head oscillation values of healthy controls and patients with IBS in each of the three conditions. The dashed line indicates a head velocity value of zero. Head oscillations are defined as the minimal value of head velocity under the zero line (undershoot), normalized by peak head velocity (see the velocity trace of the patient for the weighted condition for an illustration of head oscillation computation). Both participants showed increased head oscillations in the weighted condition, which was more pronounced in the patient.

*Post hoc* testing for group differences revealed that with strong evidence (BF_10_ = 56.4), patients had higher head oscillations in the weighted condition than healthy controls (see Table 2 for mean head oscillation values and Figure 2 for head velocity traces of all participants in the weighted condition). With anecdotal evidence (BF_10_ = 0.6), groups did not differ in the pre-condition (see Figure 3 for representative head movements over all three conditions). In the post-condition, no evidence was found for or against a group difference in head oscillations (BF_10_ = 1). We conducted further *post-hoc* tests to reveal potential differences in head oscillations between the three experimental conditions. With extreme (patients: BF_10_ = 208.1) and moderate (controls: BF_10_ = 5.1) evidence, increasing the head moment of inertia increased head oscillations. There was also extreme evidence that head oscillations decreased again after the weights had been removed (patients: BF_10_ = 443.2; controls: BF_10_ = 162.6). Importantly, with moderate evidence, head oscillations were smaller in the post- than in the pre-condition, but only in patients (BF_10_ = 5.8), not in controls (BF_10_ = 0.7).

**TABLE 2:** Head oscillation values.

	Condition		
	
Group	Pre	Weighted	Post
Patients	4.1 ± 0.66	10.4 ± 0.45	2.6 ± 0.56
Controls	2.9 ± 0.99	6.9 ± 0.56	1.6 ± 0.16

Shown are all mean head oscillation values in percent with the standard error of the mean (SEM) for all experimental conditions (within factor *weight*) for patients as well as controls (between factor *group*).

**FIGURE 4:**
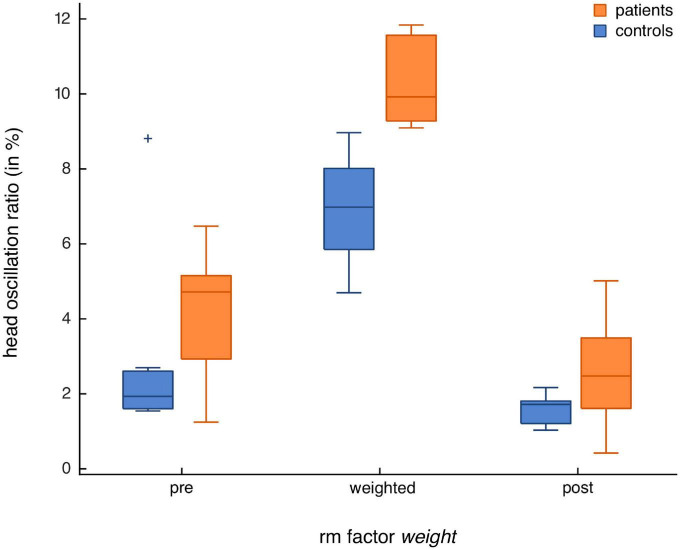
Head oscillations of healthy controls (*n* = 7) and patients with IBS (*n* = 7) for all three experimental conditions. Shown are boxplots for the repeated measures (rm) factor *weight*, with a separate box for each condition (pre, weighted, post). Results for patients are shown in orange and those for healthy controls in blue. Note the outlier in the healthy control group in the natural condition that was > 2 SD above the participants’ mean.

One healthy control showed extremely high head oscillations in the pre-condition (Figure 4), with a value ranging > 2 *SD* above participants’ mean value. Excluding this participant did not alter the direction of study results, as the model *weight* + *group* + weight*group was still the most likely model given the data (BF_10_ = 8.4 × 10^12^). However, effect sizes became notably larger for the two factors (*weight*: BF_incl_ = 4 × 10^12^; *group*: BF_incl_ = 45.1) and their interaction (BF_incl_ = 8.7). In *post hoc* testing, the head oscillation difference between patients and healthy controls in the pre-condition additionally showed moderate evidence (BF_10_ = 4), possibly because of the small inertia alterations the goggles create themselves. Differences between the pre- and the weighted condition in healthy controls altered from moderate to strong evidence (BF_10_ = 32.8).

Due to technical issues, few of the experimental rounds were performed without the control light being present (patient one: all three sessions; patient four: pre-session), using the design of our previous studies (e.g., Lehnen et al., 2019). When excluding these patients from the analysis, the results did not change (best model: *weight* + *group* + *weight* × *group*; BF_10_ = 1.1 × 10^8^), although, due to smaller sample size, BF and effect sizes became smaller when excluding these patients.

For comparability with previous studies, we also computed a frequentist rmANOVA. Results showed a significant main factor weight [*F*_(2, 24)_ = 73.7; *p* < 0.001; partial η^2^ = 0.69] and a significant main factor group [*F*_(1, 12)_ = 10.3; *p* = 0.007; partial η^2^ = 0.08]. That is, both patients and healthy controls had higher head oscillations in the weighted condition and, overall, patients had higher head oscillations than controls. The interaction *weight* × *group* did not reach statistical significance [*F*_(2, 24)_ = 3.1; *p* = 0.062]. Both groups displayed higher head oscillations in the weighted condition compared to the pre-condition (*p* < 0.001) and the post-condition (*p* < 0.001), while there was no evidence for differences in head oscillations between the pre- and the post-condition (*p* = 0.057). In line with the Bayesian analysis, excluding the healthy control with a mean oscillation value of 2 SD above the group’s mean altered the study results. In addition to the main effects [*weight*: *F*_(2, 24)_ = 119; *p* < 0.001; partial η^2^ = 0.74; *group*: *F*_(1, 17)_ = 20.8; *p* < 0.001; partial η^2^ = 0.11], the weight*group interaction became significant [*F*_(2, 24)_ = 3.6; *p* = 0.04; partial η^2^ = 0.02]. In the weighted condition, patients had increased head oscillations compared to healthy controls (*p* < 0.001). This was not the case for the pre- (*p* = 0.086) and post-condition (*p* = 1).

## Discussion

We found experimental evidence for a general transdiagnostic processing deficit in patients with IBS, who showed poorer head motor control, reflected by increased head oscillations during gaze shifts with increased head moment of inertia, compared with healthy controls. Altering the head mechanics, e.g., by increasing the head moment of inertia, introduces a mismatch between the intended and executed head movement, so that the actual sensory consequences of the head movement do not match expectations. This mismatch becomes visible in poorer head motor control, reflected by involuntary head oscillations at the end of a head movement, as head alterations are not yet incorporated in internal models of the head for sensorimotor planning. Similar to previous studies (Lehnen, 2006; Lehnen et al., 2008, 2009, 2019; Sağlam et al., 2011, 2014), in the present study, increased head inertia led to higher head oscillations in all participants. Notably, during gaze shifts under increased head moment of inertia, head oscillations of patients with IBS were more pronounced than in the healthy control group, indicating processing deficits in patients with functional somatic symptoms reaching beyond a “normal” reaction to altered head properties.

Predictive processing theory in functional somatic disorders states that persisting somatic symptoms emerge and manifest due to altered sensorimotor processing. That is, rigid expectations dominate sensory input, so that the perception of body signals becomes more and more independent from actual body states (Edwards et al., 2012; Van den Bergh et al., 2017; Henningsen et al., 2018; Pezzulo et al., 2019). In the present study, the use of such rigid expectations could explain poorer head motor control in patients with IBS under increased head inertia. The altered head properties constitute a new context that requires the use of sensory input to adapt expectations to these alterations and subsequently reduce head oscillations. If, as hypothesized, patients rely too much on expectations during sensorimotor processing, this would impair such flexible adaptation processes. Sensory signals would not be sufficiently used to “tell” the brain what is going wrong in head motor control, and, as a consequence, head oscillations would remain increased during gaze shifts under increased head inertia. In contrast, healthy controls can use sensory input to reduce head oscillations.

Increased head oscillations as a marker for impaired sensorimotor processing have also been demonstrated in patients with functional dizziness (Lehnen et al., 2019), a patient group with symptoms directly linked to gaze motor control. However, processing deficits were clearly stronger in the functional dizziness group than in patients with IBS when comparing effect sizes (effect sizes for differences to healthy controls in functional dizziness: partial η^2^ = 0.62; IBS: partial η^2^ = 0.08). This indicates that erroneous sensorimotor processing is stronger in the impaired modality, possibly playing a central role in symptom emergence and manifestation. However, as the present results show, the processing of sensorimotor signals in patients with other functional somatic symptoms (here: patients with functional gastrointestinal symptoms) is also affected in an attenuated way. These alterations might not be strong enough to present a measurable correlate for already manifested symptoms, as none of our patients with IBS reported signs of dizziness, but they may display a general impairment, putting patients at risk for developing new symptoms. Further studies investigating additional patient groups with different somatic symptom localizations should be conducted to support this speculation. Whereas measuring more patients for generalizability is certainly warranted, it is worthwhile to mention the astonishing power of the current results, reflected in an *a priori* sample size estimation of three in the affected modality and moderate to strong evidence for transdiagnostic effects with a sample size of seven.

Experimental studies specifically focusing on the interplay between expectations and sensory input within interoceptive processing of patients with IBS are still lacking. Nevertheless, many studies have investigated interoception in the gut. Patients with IBS report non-painful and painful stimuli earlier when stimulus strength is continuously increased (e.g., Ritchie, 1973; Mertz et al., 1995; Whitehead and Palsson, 1998; Verne et al., 2001; Bouin et al., 2002; Azpiroz et al., 2007; Barbara et al., 2011). This effect increases with symptom severity (Posserud et al., 2007; Simrén et al., 2018). In predictive processing, such earlier stimulus reports can be explained by overly reliant stimulus expectations that lower the stimulus strength needed for perception. Importantly, perceptual alterations in patients with IBS have also been demonstrated for other, non-visceral body locations: patients show altered responses in perceiving electrical, cold or heat stimulation of the skin on hands and feet (Bouin et al., 2001; Verne et al., 2001; Iovino et al., 2006; Zhou et al., 2010). These results are in line with our experimental findings, pointing at general, to symptom-unspecific processing alterations in patients with IBS. However, such rather subjective read-outs like reports of perceptual changes are not directly linked to the mostly unconscious perceptual processing steps in the brain and only represent a small proportion of the underlying mechanisms, i.e., the final perception. Also, such reports can underly cognitive and motivational biases that are known from research on decision making (Kahneman et al., 1982; Gilovich et al., 2002). By using a behavioral read-out that is less prone to cognitive strategies, we overcame these obstacles, providing novel evidence for altered sensorimotor processes in this group of patients with functional symptoms. These results are shown for a completely different brain circuitry, i.e., head motor control, where experimental alterations can be directly linked to information processing steps in the brain.

Interestingly, patients with IBS were able to reduce head oscillations in the post-condition, compared to the pre-condition. This difference was not found in healthy controls. A possible explanation may be that, in the pre-condition, head oscillations of healthy controls are so minor that the vestibular input generated by these oscillations is too small to drive further updating of the head plant representation in the brain. In contrast, the reduction of head oscillations in patients with IBS from pre- to post-condition could present a learning process, in which patients are able to factor in their (stronger) sensory feedback to adjust head motor planning and reduce oscillations. Such learning processes could be either driven by sufficient repetitions of experimental rounds. Alternatively, it could be that increasing the head moment of inertia provokes stronger error signals (oscillations), which enable patients with IBS to make sensory-driven updates to CNS-models and associated expectations in the weighted and subsequent post-condition. Future analysis should focus on analyzing head learning strategies in IBS patients, e.g., the effects of serial dependencies (Zimmermann, 2021). Although the exact mechanisms remain to be determined, experimental alterations like increasing the head moment of inertia might provide a promising therapeutic approach to train the flexibility of the brain when processing sensorimotor signals in different contexts. This might counteract the proposed pathophysiology in which patients over-rely on expectations vs. sensory input (Edwards et al., 2012; Van den Bergh et al., 2017; Henningsen et al., 2018; Pezzulo et al., 2019), and potentially contribute to a reduction in symptoms. Although, for IBS patients, we feel that to reduce gastrointestinal symptoms, training target should be the affected modality.

Of course, studying sensorimotor processing like in the present study constitutes one of many possible ways to look at pathophysiological mechanisms in IBS in a rather specialized, neuroscientific framework. For instance, there is also impressive research on the role of gut mucosa, inflammatory and immune processes (Enck et al., 2016) in an attempt to capture more thoroughly the pathophysiology in IBS and related functional gastrointestinal symptoms. However, looking at central processing of body signals in IBS and functional somatic disorders in general is promising, as it provides a unifying framework for the emergence and manifestation of many different types of somatic symptoms across functional somatic disorders. This helps to define functional somatic disorders with positive diagnostic criteria, based on measurable correlates of functional somatic symptoms. Furthermore, sensorimotor processing deficits can exist and be measured in a dimensional way, demonstrating graded effects in patients with functional dizziness or IBS and might therefore strengthen a dimensional understanding of pathophysiology. Nevertheless, it remains to be seen how alterations in sensorimotor processing affect patients’ subjective experience and which factors determine the manifestation of a symptom.

In summary, our results provide evidence for a general, symptom-unspecific, transdiagnostic central processing deficit in functional somatic disorders. In a gaze shift paradigm, patients with IBS showed more pronounced head oscillations during eye head gaze shifts toward visual targets under increased head moment of inertia than healthy controls. This was similar to patients with functional dizziness, but less pronounced (Lehnen et al., 2019). These findings indicate an impaired interplay between expectations and sensory input in sensorimotor processing across functional somatic symptoms and supports the predictive processing account of functional somatic disorders (Edwards et al., 2012; Van den Bergh et al., 2017; Henningsen et al., 2018; Pezzulo et al., 2019). Moreover, these findings contribute to a unified and dimensional understanding of the pathophysiology of functional somatic symptoms and disorders and might help in developing further diagnostic and treatment approaches in this patient group.

## Data availability statement

The original contributions presented in this study are publicly available and can be accessed on the manuscript’s Open Science Framework (OSF) webpage under the following link: https://osf.io/cnu36/.

## Ethics statement

The studies involving human participants were reviewed and approved by the Ethics Committee of the Technical University of Munich. The patients/participants provided their written informed consent to participate in this study.

## Author contributions

LS, SW, RvK, and NL designed the study. JH and DP gave input on IBS specifics. LS, FR, and AH collected the data. LS, FR, AH, SG, and NL analyzed and interpreted the data. LS created the figures and wrote the initial manuscript. All authors revised the manuscript critically for important intellectual content and gave final approval of the version to be published.

## Footnotes

1
https://jasp-stats.org

## Abbreviations

CO_2_: Carbon dioxide
IBS: Irritable Bowel Syndrome
ICD-10: 10th version of the International Statistical Classification of Diseases and Related Health Problems
DSM-5: Diagnostic and Statistical Manual of Mental Disorders, Version 5
SCID-5-CV: Structural Clinical Interview for DSM-5—Clinician Version
vHIT: video-assisted Head Impulse Test
LED: Light Emitting Diode
rmANOVA: repeated measures Analysis of Variance
BF: Bayes Factor.
